# Key Lessons and Impact of the Growing Healthy mHealth Program on Milk Feeding, Timing of Introduction of Solids, and Infant Growth: Quasi-Experimental Study

**DOI:** 10.2196/mhealth.9040

**Published:** 2018-04-19

**Authors:** Rachel A Laws, Elizabeth A Denney-Wilson, Sarah Taki, Catherine G Russell, Miaobing Zheng, Eloise-Kate Litterbach, Kok-Leong Ong, Sharyn J Lymer, Rosalind Elliott, Karen J Campbell

**Affiliations:** ^1^ Institute for Physical Activity and Nutrition School of Exercise and Nutrition Sciences Deakin University Geelong Australia; ^2^ Centre for Obesity Management and Prevention Research Excellence in Primary Health Care Sydney Australia; ^3^ School of Nursing University of Sydney Sydney Australia; ^4^ Sydney Local Health District Sydney Australia; ^5^ Faculty of Health University of Technology Sydney Sydney Australia; ^6^ La Trobe Analytics Lab La Trobe University Melbourne Australia; ^7^ Faculty of Pharmacy University of Sydney Sydney Australia

**Keywords:** mHealth, obesity prevention, infancy, parents, breastfeeding, complementary feeding, formula feeding

## Abstract

**Background:**

The first year of life is an important window to initiate healthy infant feeding practices to promote healthy growth. Interventions delivered by mobile phone (mHealth) provide a novel approach for reaching parents; however, little is known about the effectiveness of mHealth for child obesity prevention.

**Objective:**

The objective of this study was to determine the feasibility and effectiveness of an mHealth obesity prevention intervention in terms of reach, acceptability, and impact on key infant feeding outcomes.

**Methods:**

A quasi-experimental study was conducted with an mHealth intervention group (Growing healthy) and a nonrandomized comparison group (Baby’s First Food). The intervention group received access to a free app and website containing information on infant feeding, sleep and settling, and general support for parents with infants aged 0 to 9 months. App-generated notifications directed parents to age-and feeding-specific content within the app. Both groups completed Web-based surveys when infants were less than 3 months old (T1), at 6 months of age (T2), and 9 months of age (T3). Survival analysis was used to examine the duration of any breastfeeding and formula introduction, and cox proportional hazard regression was performed to examine the hazard ratio for ceasing breast feeding between the two groups. Multivariate logistic regression with adjustment for a range of child and parental factors was used to compare the exclusive breastfeeding, formula feeding behaviors, and timing of solid introduction between the 2 groups. Mixed effect polynomial regression models were performed to examine the group differences in growth trajectory from birth to T3.

**Results:**

A total of 909 parents initiated the enrollment process, and a final sample of 645 parents (Growing healthy=301, Baby’s First Food=344) met the eligibility criteria. Most mothers were Australian born and just under half had completed a university education. Retention of participants was high (80.3%, 518/645) in both groups. Most parents (226/260, 86.9%) downloaded and used the app; however, usage declined over time. There was a high level of satisfaction with the program, with 86.1% (143/166) reporting that they trusted the information in the app and 84.6% (170/201) claiming that they would recommend it to a friend. However, some technical problems were encountered with just over a quarter of parents reporting that the app failed to work at times. There were no significant differences between groups in any of the target behaviors. Growth trajectories also did not differ between the 2 groups.

**Conclusions:**

An mHealth intervention using a smartphone app to promote healthy infant feeding behaviors is a feasible and acceptable mode for delivering obesity prevention intervention to parents; however, app usage declined over time. Learnings from this study will be used to further enhance the program so as to improve its potential for changing infant feeding behaviors.

## Introduction

The World Health Organization has identified the prevention of obesity in early life as a key priority [1]. Children are becoming overweight at a relatively young age, with 22.8% of Australian children aged 2 to 4 years already overweight or obese [2] with substantial health and economic consequences [3]. Infants who are at the highest end of the distribution for body mass index (BMI) or who grow rapidly during infancy are at increased risk of subsequent obesity in both childhood and adulthood [4,5].

Infant feeding practices, including the duration of breastfeeding, formula feeding practices [6-8], when solid food is introduced [9], and whether a baby is predominantly fed on a schedule or according to their hunger and satiety cues, are associated with rapid weight gain [10,11]. Australian and international infant feeding guidelines recommend that infants are exclusively breastfed to around 6 months of age when solid foods should be introduced and that breastfeeding continue for 12 months or longer [12,13]. However, Australian data indicate that only 15% of infants are exclusively breastfed until 6 months of age, with 40% of infants having at least some formula by 1 month of age [14]. Similar numbers are found in the United States where national rates of exclusive breastfeeding at 6 months are 22% [15]. Furthermore, over a quarter (28.4%) of Australian infants are introduced to solids by 4 months of age and over half (56.2%) by 5 months [14]. This clearly highlights the need for interventions to promote recommended infant feeding practices.

There is increasing evidence that children from low socioeconomic backgrounds have higher rates of overweight and obesity [16], and socioeconomic disparities begin early in life [17]. A recent review found that a strong socioeconomic gradient exists for the majority of early life risk factors for child obesity [18], suggesting that early intervention is critical in reducing socioeconomic inequalities in overweight and obesity in childhood and related chronic diseases in adulthood. However, socioeconomically disadvantaged families are often more difficult to reach and may be less likely to participate in traditional programs that support healthy behaviors [19].

One emerging and promising area to facilitate parent engagement at a low cost is the provision of support for parents through electronic media such as the Internet or smartphones. Smartphone ownership is increasing worldwide, with Australians having the highest rate (93%) of access to smartphones [20]. Women aged 18 to 49 years (many of whom are mothers) spend on average 21 hours a week on their smartphone [21]. Well-designed smartphone apps can provide around the clock high-quality information, as well as personalized and tailored support at low cost [22]. Evidence suggests that although parents increasingly rely on the Internet for information on infant feeding and care [23-27], less research has been conducted on the use of smartphone apps in the postpartum period. One study reported that low-income women commonly use apps during pregnancy, but not in the postpartum period because of the limited availability of high-quality apps, creating a postpartum app gap [28]. This is in line with our own research where we found that infant feeding apps available in Australia are generally of low quality [29].

Early research on the effectiveness of mHealth interventions in changing health behavior is promising [30-32]; however, this is the first study, to our knowledge, to investigate the effectiveness of mHealth interventions in influencing parents’ infant feeding behaviors. The Growing healthy (GH) study aimed to explore the feasibility of providing information and support to parents for healthy infant feeding practices using an mHealth program. This paper reports on the effectiveness of GH in terms of reach, use, acceptability, and several key infant feeding outcomes including the promotion of exclusive or continued breastfeeding, best practice formula feeding, timing of introduction of solids, and infant growth.

## Methods

The study utilized a quasi-experimental design with an mHealth intervention group and a concurrent nonrandomized comparison group. A detailed description of the development of the GH program and the methods of the feasibility study has been previously published [33]. Key components related to this paper are described below.

### Study Participants

The eligibility criteria for participation in the intervention group (GH) included being pregnant (30+ weeks gestation) or parent or main carer of an infant younger than 3 months, ownership of any type of mobile phone, ability to speak and read English, age 18 years or older, and residing in Australia. Participants were recruited using 3 methods: via their primary care providers in socioeconomically disadvantaged communities in 2 Australian states; face-to-face by researchers in first-time parent groups; or Web-based advertising. A concurrent nonrandomized comparison group (Baby’s First Food, BFF) was recruited via online forums, social networking sites, and blogs and received usual care. The eligibility criteria for participation were the same as the intervention arm with the exception that participants were not required to own a mobile phone. Enrollment to both groups of the study involved the completion of a Web-based screening form, a consent form, and a baseline survey. Further details of the recruitment process and outcomes have been published elsewhere [34].

### The Growing Healthy Program

In brief, the GH program aimed to encourage parents to engage in infant feeding practices that promote healthy rather than excess weight gain, with a focus on socioeconomically disadvantaged parents. The aims of the program were as follows:

- Promote breastfeeding.
- If breastfeeding was not possible, promote best practice formula feeding.
- Delay the introduction of solids to around 6 months of age but not before 4 months.
- Promote healthy first foods.
- Promote healthy infant feeding practices (including feeding to appetite, repeated neutral exposure to healthy food, and avoiding using food as a reward).
- Optimize infant dietary exposure to fruits and vegetables.

The main delivery media for the program were an app and website, which provided parents with evidence-based article and videos containing practical advice and strategies consistent with national guidelines on infant feeding from birth until 9 months of age. The development of the program was guided by the Behaviour Change Wheel and the Capability, Opportunity and Motivation model of behavior change [35]. For each program aim (target behavior), key determinants were identified using prior formative work [36,37] and literature and mapped to intervention strategies. Participants received 3 personalized push notifications or text messages (short message service, SMS) per week targeting specific intervention strategies and behavior change techniques as detailed in our protocol paper [33]. Push notifications were also tailored to each infant’s age and stage of development as well as their feeding mode (breast, formula, or mixed feeding), directing them to relevant content in the app. A weekly email was also sent that included the 3 messages for the week with links to the website. This was introduced part way through the intervention in response to the low number of push notifications being opened. Participants were also invited to join a Facebook group where one feeding message per week was posted by a moderator, and participants were encouraged to discuss practical experiences around infant feeding.

### Data Collection

Data were collected at 3 time points via a Web-based survey: when infants were less than 3 months old (T1), when the infant was 6 months of age (T2), and when the infant was 9 months of age (T3). To compensate participants for the time involved in completing surveys, GH participants received a gift voucher worth Aus $20 per survey completed, and BFF participants received Aus $40 for the completion of 2 or more surveys. Nonresponders to the survey were sent 3 email reminders, 1 week apart. We also collected data from analytics within the app.

#### Assessment of Breastfeeding Duration and Exclusivity

At T1, T2, and T3, parents were asked to report if their infant was currently breastfed and the infant’s age in weeks when breastfeeding ceased if they were no longer breastfeeding. At T3, they were also asked about what they were feeding their infant: (1) breast milk, solids, and water or juices; (2) infant formula, solids, and water or juices; or (3) a combination of breast milk, infant formula, solids, and water or juices. Exclusive breastfeeding at T2 was determined by the question “Does baby have other fluids or food apart from breast milk?” All of those participants who were breastfeeding were asked additional questions at T1 and T2 about introduction of infant formula and the child’s age at introduction of formula.

#### Assessment of Best Practice Formula Feeding

Formula preparation was assessed at each time point using a valid and reliable questionnaire [38] including the following items: follows instructions on the tin for loosely packed level scoops, adds water to the bottle first, and never adds more formula than the specifications on the tin. Additional questions about formula feeding practices were asked at each time point, including whether cereal was added to the bottle to ensure baby slept longer or stayed fuller longer, whether participants held their baby when feeding with a bottle, whether participants believed it was important for the baby to finish all the formula in the bottle, and whether participants allowed their baby’s appetite to guide feeding. These questions were taken from the previously validated infant feeding questionnaire [39].

#### Assessment of Timing of Solid Introduction

At T1 and T2, parents were asked whether solids had been introduced and, if so, the infant’s age in weeks at introduction.

#### Assessment of Child Anthropometrics

At each time point, the parent was asked to provide the most recent weight and length data from their infant’s health record.

#### Assessment of Demographic Characteristics

The infant’s sociodemographic characteristics including age, gender, birth order, and whether infants were Aboriginal or Torres Strait Islander were collected in the survey at T1. Also collected at T1 were parental characteristics, including primary carer’s age, country of birth, relationship status, self-rated health, employment status, education level, and annual household income.

#### Assessment of App Usage

Participants’ app usage was extracted from the GH activity log hosted on the Azure cloud in Southeast Australia. The key metrics collected included number of pages viewed per session (1 session=each day they accessed the app), number of sessions from the point participants activated the app until 9 months of the infant’s age, and the number of push notifications opened. Furthermore, participants’ device type (Android/iPhone) was also collected.

#### Assessment of App Acceptability

At T3, participants were asked 25 questions relating to feasibility, acceptability, ease of use, and perceived usefulness of the app (and website) and the program overall. These survey questions were adapted from EMPOWER, an Australian mHealth intervention aimed at weight loss in adults [40], and the app quality assessment tool [29].

### Statistical Analysis

Basic descriptive analysis and cross-tabulations were calculated. Baseline characteristic comparisons were made using chi-square test and *t* test as appropriate to the variable type. Survival analysis was used to assess the difference in timing of feeding practices, including duration of any breastfeeding, timing of introduction of infant formula, and timing of solids introduction between the 2 groups—GH and BFF. Kaplan-Meier survival curves were used to assess the mean and median of breastfeeding duration and the timing of introduction of solids. Differences between the groups were assessed using Breslow test. Those cases where the child was reported as still being breastfed at T3 were classed as censored observations in the breastfeeding duration analysis. Cox proportional hazards regression models in the analysis of breastfeeding duration and time to solids introduction were used to account for covariates such as child’s gender, whether first born, dummy use, maternal smoking status, mother’s country of birth, parental education and work status, household income, and maternal age and prepregnancy BMI. Mother’s self-rated health and possession of a health card were considered but due to high correlation with other covariates were excluded from the final analysis. Evaluation of the log-minus-log survival curves provided no evidence that the assumptions of proportional hazards were not met.

Multivariate logistic regression was used in the analysis of differences between GH and BFF for binary outcomes, including proportion exclusively breastfeeding; formula feeding outcomes; and proportion who had introduced solids before 4 months, after 6 months, and at 4, 4.5, 5, 5.5, and 6 months.

Differences between GH and BFF group continuous variables, which included child BMI z-score, weight, and length at time points between birth and T3, were assessed using mixed effect polynomial regression models with an unstructured covariance structure. A random intercept and a random slope for age to allow individual growth rates were fitted. Quadratic and cubic age variables to model nonlinear growth were included. Covariates included in the model were child’s gender, whether first born, dummy use, maternal smoking status, mother’s country of birth, parental education and work status, household income, and maternal age and prepregnancy BMI. All statistical analysis was performed using IBM Corporation SPSS version 24 [41].

## Results

### Study Participants

**Figure 1:**
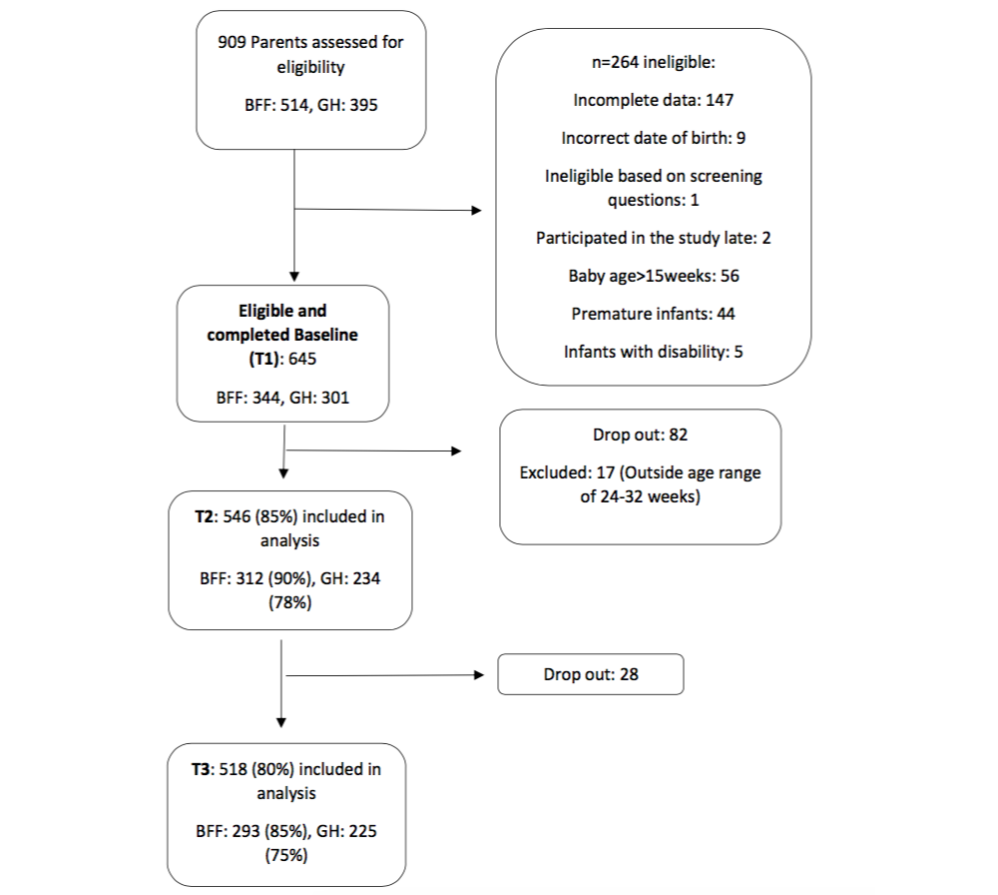
Flowchart detailing study participants.

A total of 909 subjects commenced enrollment into the study; however, 264 were ineligible predominately because they did not complete the baseline survey or their baby was older than 15 weeks or born prematurely (Figure 1). The final sample included 645 carer/child dyads at baseline (GH: 301; BFF: 344). Retention to the study was high at T2 (84.7%) and T3 (80.3%; Figure 1).

### Baseline Characteristics

At enrollment, the mean age of the children in the GH group was significantly younger than the BFF group (7.0 weeks compared with 7.9 weeks). Furthermore, the GH group compared with the BFF group contained a significantly higher proportion of first-born children, with GH mothers being younger (30.4 years compared with 31.2 years), a lower proportion of GH mothers being Australian born (84.1% vs 90.1%), and a lower proportion in the highest household income category (29.4% vs 36.8%). GH also contained a lower proportion of breastfeeding mothers and higher proportion of formula and mixed feeding mothers at baseline when compared with BFF (*P*<.001). Other characteristics considered were not found to be significantly different at baseline between the 2 groups. Details of the baseline characteristics of study participants are provided in Table 1.

### App Usage

**Figure 2:**
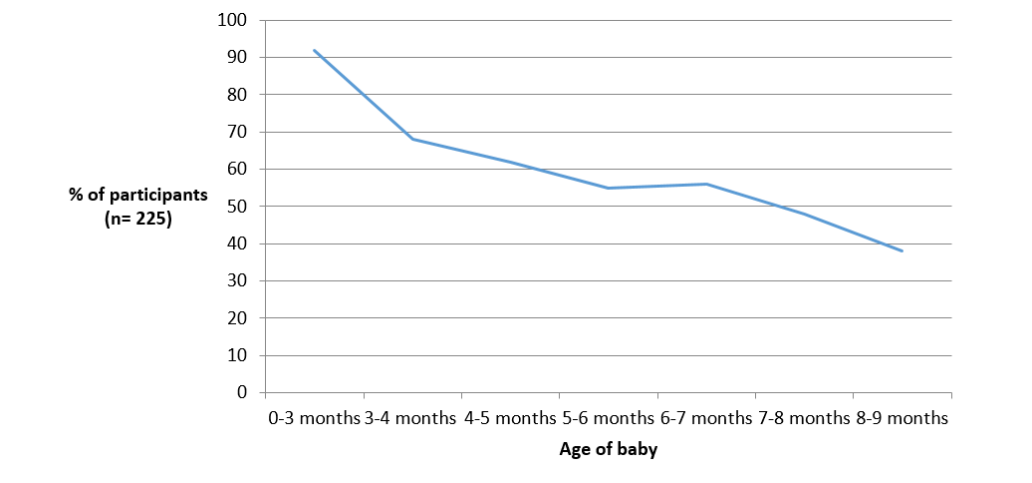
The percentage of participants who used the Growing healthy app throughout the 9-month program.

**Figure 3:**
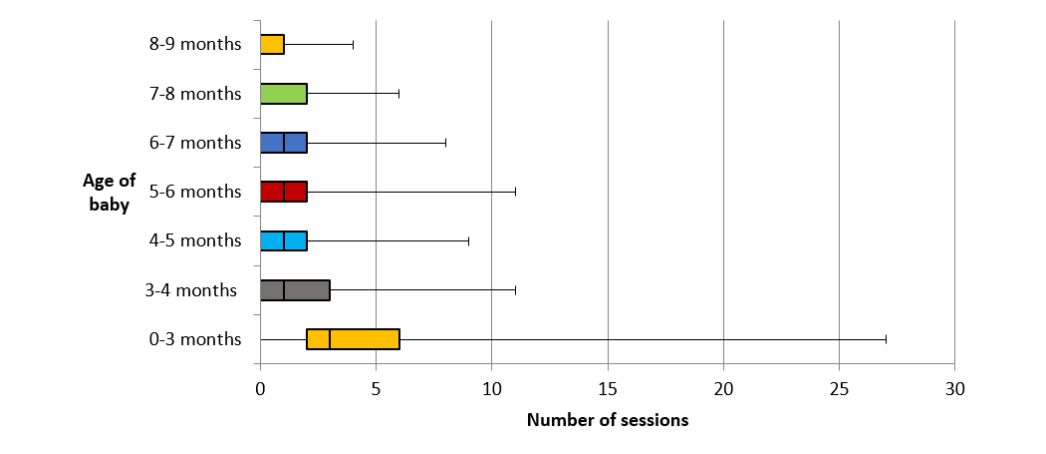
Participants’ frequency of using the Growing healthy app throughout the 9-month program.

Of the 301 participants, 260 (86.4%) opted to access the program via the app, and 41 (13.6%) via the website/SMS. App participants were provided with a code to enable them to download the app from either Google Play or the App Store, and 74.8% (225/301) of participants downloaded the app. More than half of the sample used iPhones (71.6%), with 28.4% using Android phones and 11.9% website and SMS. Of app users, Android phone usage was higher among non-university-educated participants (31.7%) compared with university-educated participants (24.5%). App users were sent 3 push notifications each week, and on average, 11 push notifications were opened over the time of the study (8.0% of all notifications). App usage declined over time from 92.0% using the app at least once on enrollment to 38.2% at study completion when infants were aged 8 to 9 months of age (Figure 2), with a similar decline in the mean number of sessions using the app across the duration of the study (Figure 3).

**Table 1:** Baseline characteristics of study participants by intervention group.

Characteristics			Growing healthy (n=301)	Baby’s First Food (n=344)	*P* value
**Child factors**					
	Age (weeks)		7.0 (3.7)	7.9 (3.8)	.001
	**Gender, n (%)**				
		Boys	150 (49.8)	167 (48.5)	.74
		Girls	151 (50.2)	177 (51.5)	
	**Aboriginality, n (%)**				
		Nonaboriginal nor Torres Strait Islanders	294 (97.7)	335 (97.4)	.81
		Aboriginal and/or Torres Strait Islanders	7(2.3)	9 (2.6)	
	First-born baby, n (%)		173 (57.5)	133 (38.7)	<.001
	Dummy use at baseline, n (%)		164 (54.5)	163 (47.4)	.07
**Parental factors**					
	Mother’s age, years, mean (SD)		30.4 (4.7)	31.2 (4.4)	.04
	Mother prepregnancy body mass index, kg/m^2,^ mean (SD)		26.6 (5.7)	27.2 (6.8)	.23
	Maternal current smoking status, n ( %)		18 (6.0)	15 (4.4)	.35
	Maternal country of birth—Australian born, n (%)		253 (84.1)	310 (90.1)	.02
	Relationship status—married, n (%)		289 (96.0)	332 (96.5)	.74
	Health care card holder, n (%)		48 (15.9)	53 (15.4)	.85
	**Maternal self-rated health, n (%)**				
		Poor/fair	30 (10.0)	28 (8.1)	.51
		Good	116 (38.5)	152 (44.2)	
		Very good	124 (41.2)	131 (38.1)	
		Excellent	31 (10.3)	33 (9.6)	
	**Maternal education, n (%)**		**n=289**	**n=332**	
		Low	61 (21.1)	56 (16.4)	.29
		Medium	88 (30.5)	115 (33.6)	
		High	140 (48.4)	171 (60.0)	
	**Maternal working status, n (%)**		**n=301**	**n=342**	
		Not working	261 (86.7)	298 (86.6)	.87
		Working	40 (13.3)	44 (12.8)	
	**Paternal education, n (%)**		**n=289**	**n=332**	
		Low	56 (19.4)	64 (19.3)	.56
		Medium	144 (49.8)	153 (46.1)	
		High	89 (30.8)	115 (34.6)	
	**Paternal working status, n (%)**		**n=289**	**n=331**	
		Not working	12 (4.2)	7 (2.1)	.14
		Working	277 (95.8)	324 (97.9)	
	**Annual house income, Aus $, n (%)**		**n=301**	**n=288**	
		≤51,999	35 (13.7)	44 (15.3)	.02
		52,000-77,999	79 (31.0)	57 (19.8)	
		78,000-99,999	81 (25.9)	66 (28.1)	
		100,000 or more	75 (29.4)	106 (36.8)	
	**Feeding groups, n (%)**				
		Breastfeeding	196 (65.1)	245 (71.2)	<.001
		Formula feeding	52 (17.3)	48 (14.0)	
		Mixed feeding	53 (17.6)	51 (14.8)	

### Acceptability

Overall, participants reported high levels of satisfaction with the program, with 88.1% agreeing that they liked the program and 84.6% informing that they would recommend it to a friend (Table 2). Most parents (86.1%) reported that the app provided trustworthy information and was easy to understand (91.0%) and use (78.3%), with less than 5% of parents expressing concern about data usage when using the app. However, just over a quarter of parents reported that the app failed to work at times. Nearly 20% of participants who completed T3 reported disabling push notifications on their phone. For those receiving push notifications, a majority found them helpful, well suited to their baby’s age and stage of development, and appropriate in terms of number and timing of messages. However, just over a third reported that the messages disappeared before they read them, and nearly 40% were unsure how to retrieve push notifications once they had disappeared from the screen.

**Table 2:** Participant satisfaction with the Growing healthy program.

Satisfaction item		Agree or strongly agree, n (%)
**Overall program (n=201)**		
	Overall I liked the Growing healthy program	177 (88.1)
	I would recommend the Growing healthy program to a friend	170 (84.6)
	Covered all I needed on feeding	134 (66.7)
**Growing healthy app (n=166)**		
	Using the app was an enjoyable experience	125 (75.3)
	I can trust the information in the app	143 (86.1)
	The app did everything I expected it to do	128 (77.1)
	I liked the layout/look of the app	131 (78.9)
	The language used in the app was easy to understand	151 (91.0)
	I found the Growing healthy app easy to use	130 (78.3)
	I was concerned about data usage/costs when using the app	7 (4.2)
	Hard to navigate	20 (12.0)
	The Growing healthy app failed to work at times, n=166	43 (25.9)
**Push notifications (n=126)**		
	Push notifications often disappeared before I had a chance to open them	43 (34.1)
	I did not know how to retrieve push notifications once they disappeared from the screen	50 (39.7)
	I would prefer to receive text messages than push notifications	37 (29.4)
**Push notifications and text messages (n=201)**		
	I was happy with the number of push notifications/texts received each week	127 (63.2)
	I was happy with the timing of push notifications/texts	134 (66.7)
	I found the push notifications/texts helpful	127 (63.2)
	I found the push notifications suited my baby’s age and stage of development	138 (68.7)

### Feeding Outcomes

#### Breastfeeding

**Figure 4:**
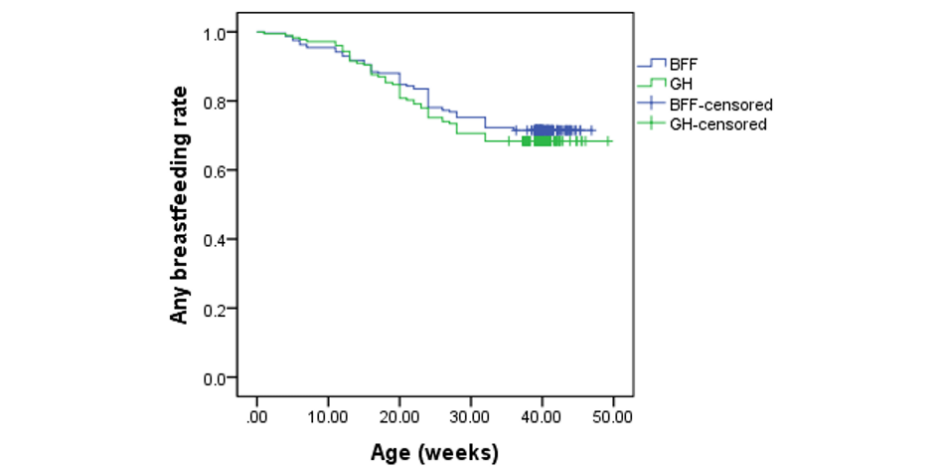
Rates of any breastfeeding duration by intervention group (BFF: Baby’s First Food; GH: Growing healthy).

The percentage of breastfeeding and mixed feeding mothers who reported having stopped breastfeeding by T3 was 31.6% and 28.5% for GH and BFF, respectively (Figure 4). The mean duration of any breastfeeding at T3 for GH was 39.6 weeks (95% CI 37.5-41.8) compared with 39.0 weeks (95% CI 37.3-40.7) for BFF. There was no statistically significant difference in the mean duration of any breastfeeding between the 2 groups (*P*=.46). The hazard ratio for ceasing any breastfeeding in GH compared with BFF was not significantly different (hazard ratio 1.13; 95% CI 0.74-1.74; *P*=.57). Stratified analysis by whether child first born (first-time vs non-first-time mothers) suggested that duration of any breastfeeding were not significantly different between GH and BFF, regardless of whether the child was first born or not. However, across both groups, the median duration of any breastfeeding for non-first-time mothers (41.9 weeks) were significantly longer than that of first-time mothers (37 weeks) (*P*=.02). Cox hazard regression also revealed that first-time mothers were more likely to cease breastfeeding at T3 when compared with non-first-time mothers (hazard ratio 1.63; 95% CI 1.06-2.52; *P*=.03). For maternal education, no differential effects on any breastfeeding duration were found.

The proportion of breastfeeding mothers who were exclusively breastfeeding at baseline was 84% (GH) and 83% (BFF). At 6 months, the proportion of exclusive breastfeeding among the GH and BFF groups was 9% and 13%, respectively, which after adjustment for covariates showed no statistically significant difference between the groups (adjusted odds ratio, AOR 1.25; 95% CI 0.46-3.32; *P*=.65). Among exclusive breastfeeding mothers, the mean duration of any breastfeeding was also similar between the 2 groups, with a mean duration of any breastfeeding for GH of 43.4 weeks (95% CI 41.4-45.4) and for BFF of 41.0 weeks (95% CI 39.7-42.4).

#### Formula Feeding

At all 3 time points, there was a trend for a higher proportion of GH participants preparing formula correctly compared with BFF group (Table 3). After adjusting for all covariates, formula preparation practice was not significantly different between groups (AOR 1.00; 95% CI 0.48-2.10) at baseline. At T2 and T3, GH participants had slightly higher odds of preparing formula correctly in comparison with BFF (AOR of 1.25 at T2 and 1.67 at T3); however, neither was statistically significant. Most participants (99%) from both GH and BFF did not add cereal to bottle during formula preparation (data not shown). At T3, parents in the GH group were less likely to hold their baby when giving a bottle compared with mothers in the BFF group (odds ratio 0.45, 95% CI 0.20-0.95). At T2, but not baseline or T3, a higher proportion of mothers in GH than that of BFF believed that it is important to finish all formula in the bottle (AOR 2.65, 95% CI 1.04-6.71). No significant difference was found for other formula feeding behaviors such as parents’ attitude toward letting baby’s appetite guide feeding (Table 3).

#### Introduction of Solid Food

The median age for solid introduction was 21.0 weeks for both GH and BFF (GH: 95% CI 20.4-21.6; BFF: 95% CI 20.3-21.6). There was not a statistically significant difference in hazard rates for solid introduction timing between the 2 groups (hazard ratio 0.946; 95% CI 0.76-1.18). No statistically significant difference was found in the proportion introducing solids at different ages between GH and BFF groups (Table 3). Parents in the GH group were less likely to introduce solids before 4.5 months when compared with those in the BFF group (AOR 0.54-0.66); however, statistical significance was not reached (*P*=.09). Few babies in either the GH or the BFF group received solid food before 4 months (Table 4).

#### Infant Growth Trajectories

Pairwise comparisons of predicted child BMI z-score, weight, and length at each time point from the mixed effect polynomial regression model between GH and BFF are shown in Table 5. BMI z-score of GH children from birth to T3 were similar to those in the BFF group (*P* ≥.05). GH children compared with BFF children had lower weight from T1 to T3, but the mean difference was small (0.12-0.32 kg). Similarly, the height of GH children was also slightly shorter than BFF children from T1 to T3 (mean difference 0.47-1.11 cm). Growth trajectories of BMI z-score, weight, and length were not significantly different between GH and BFF (*P*>.05; Multimedia Appendix 1).

**Table 3:** Comparison of infant feeding practices by intervention group.

Variables			Intervention (Growing healthy)	Control (Baby’s First Food)		Total		Adjusted odds ratio^a^ (95% CI)		*P* value	
**Exclusive breastfeeding**											
	**T1, n**		195	246		441					
		Yes, n (%)	164 (84.1)		202 (82.1)		366 (82.9)		1.37 (0.72-2.66)		.34
	**T2, n**		111	160		271					
		Yes, n (%)	10 (9.0)		20 (12.5)		31 (11.4)		1.22 (0.46-3.32)		.69
**Prepared formula correctly**											
	**T1, n**		105	99		204					
		Yes, n (%)	48 (45.7)		38 (38.4)		86 (42.2)		1.00 (0.48-2.10)		>.99.
	**T2, n**		101	116		217					
		Yes, n (%)	58 (57.4)		56 (48.3)		114 (52.5)		1.25 (0.64-2.44)		.52
	**T3, n**		108	137		245					
		Yes, n (%)	66 (61.1)		71 (51.8)		137 (55.9)		1.67 (0.87-3.20)		.13
**Held baby when giving a bottle**											
	**T1, n**		105	99		204					
		Yes, n (%)	103 (98.1)		96 (97.0)		199 (97.5)		1.71 (0.28-10.64)		.57
	**T2 (N=217) n**		101	116							
		Yes, n (%)	87 (86.1)		103 (88.8)		190 (87.6)		0.73 (0.27-1.97)		.54
	**T3, n**		115	137		252					
		Yes, n (%)	73 (63.5)		102 (74.5)		175 (69.4)		0.48 (0.23-1.00)		.05
**Important to finish all formula in the bottle**											
	**T1, n**		105	99		204					
		Yes, n (%)	20 (19.1)		24 (24.2)		44 (21.6)		0.72 (0.3-1.69)		.45
	**T2, n**		n=101	n=116		217					
		Yes, n (%)	26 (25.7)		15 (12.9)		41 (18.9)		2.65 (1.04-6.71)		.04
	**T3, n**		n=115	n=121		252					
		Yes, n (%)	21 (18.1)		16 (13.2)		37 (14.7)		1.60 (0.66-3.88)		.30
**Let baby’s appetite guide feeding**											
	**T1, n**		53	51		104					
		Yes, n (%)	46 (86.8)		44 (86.3)		90 (86.5)		0.87 (0.12-6.48)		.89
	**T2, n**		n=30	n=33		63					
		Yes, n (%)	22 (73.3)		23 (69.7)		45 (71.4)		2.32 (0.29-18.24)		.43

^a^Logistic regression adjusted for child age, gender, whether first born, dummy use, maternal age, smoking status, country of birth, maternal and paternal education, maternal and paternal working status, maternal prepregnancy body mass index, and house income.

**Table 4:** Comparison of age at which solid foods were introduced by intervention group.

Variables		Total, (n=481)	Intervention (Growing healthy), n=208	Control (Baby’s First Food), n=273	Adjusted odds ratio^a^ (95% CI)	*P* value
**Age at which solids were introduced**						
	Before 4 months (0-15 weeks), n (%)	21 (4.4)	10 (4.8)	11 (4.0)	0.46 (0.12-1.77)	.26
	At 4 months (0-16 weeks), n (%)	64 (13.3)	28 (13.5)	36 (13.2)	0.54 (0.26-1.09)	.09
	At 4.5 months (0-18 weeks), n (%)	134 (27.9)	56 (26.9)	78 (28.6)	0.63 (0.38-1.06)	.08
	At 5 months (0-20 weeks)	234 (48.6)	104 (50.0)	130 (47.6%)	0.92 (0.58-1.44)	.70
	At 5.5 months (0-22 weeks), n (%)	304 (63.2)	145 (69.7)	159 (58.2)	1.42 (0.89-2.26)	.14
	At 6 months (0-24 weeks), n (%)	409 (85.0)	184 (88.5)	225 (82.4)	1.14(0.61-2.11)	.68
	After 6 months (0-25 weeks), n (%)	433 (90.0)	194 (93.3)	239 (87.5)	1.22(0.57-2.62)	.61

^a^Logistic regression adjusted for child age, gender, whether first born, dummy use, maternal age, smoking status, country of birth, maternal and paternal education, maternal and paternal working status, maternal prepregnancy body mass index, and house income.

**Table 5:** Predicted mean child body mass index (BMI) z-score, weight, and length of Growing healthy (GH) and Baby’s First Food (BFF) at birth, 3 months (T1), 6 months (T2), and 9 months (T3).

Anthropometry		Growing healthy, predicted mean^a^ (95% CI)	Baby’s First Food, predicted mean^a^ (95% CI)	*P* value
**BMI^b^** **z-score**				
	Birth	0.22 (0.22-0.34)	0.33 (0.22-0.44)	.21
	T1	1.57 (1.45-1.69)	1.71 (1.59-1.83)	.11
	T2	0.44 (0.31-0.57	0.26 (0.14-0.38)	.05
	T3	0.24 (0.09-0.38	0.28 (0.16-0.41)	.61
**Weight**				
	Birth	3.49 (3.43-3.56)	3.55 (3.48-3.61)	.26
	T1	4.62 (4.55-4.69)	4.76 (4.70-4.82)	.003
	T2	7.23 (7.15-7.30)	7.55 (7.48-7.61)	<.001
	T3	8.62 (8.54-8.70)	8.78 (8.71-8.85)	.003
**Length**				
	Birth	50.55 (50.28-50.82)	50.71 (50.46-50.97)	.39
	T1	54.97 (54.70-55.25)	55.51 (55.25-55.77)	.01
	T2	64.97 (64.67-65.27)	66.08 (65.81-66.36)	<.001
	T3	69.78 (69.46-70.10)	70.25 (69.97-70.53)	.03

^a^Predicted means derived from mixed effect polynomial regression model with adjustment for child age, gender, whether first born, dummy use, maternal age, smoking status, country of birth, maternal and paternal education, maternal and paternal working status, maternal prepregnancy body mass index, and household income.

^b^BMI: body mass index.

## Discussion

### Principal Findings

This is the first study, to our knowledge, to report on the feasibility and effectiveness of an mHealth intervention for prevention of obesity in infancy. Our findings support the use of mHealth as a feasible and acceptable mode of delivery of an intervention targeting parents’ infant feeding behaviors due to participants’ high levels of reported satisfaction and retention to the program, despite some technical difficulties using the app and decline in engagement over time. We were, however, unable to demonstrate any impact of the intervention on the target behaviors and outcomes of breastfeeding duration or exclusivity, timing of introduction of solids, or infant growth trajectories. The findings suggest that the GH intervention may have positively impacted on formula preparation practices, although this was not significant in fully adjusted models and requires confirmation in an adequately powered randomized controlled trial.

The findings of this study support mHealth as an acceptable mode of delivery for obesity prevention interventions in infancy, particularly those targeting infant feeding, with high rates of recruitment [42] and a retention rate of 80% at 9 months follow-up. It is important to acknowledge that the high retention rates might reflect, in part, the payment offered for survey completion and the use of 3 reminders. Acceptability of the program is supported by the high rates of reported user satisfaction with the program. This is consistent with findings from our qualitative follow-up interviews with parents [43] where they reported engagement with the program was promoted by the credibility of the program source, the user-friendly interface, and tailoring of content and push notifications to the baby’s age and key transition points. Our findings are congruent with existing research, which suggests that parents are increasingly relying on online source of information for infant feeding and care [23-27].

However, a number of factors may have reduced engagement with the program. Over a quarter of participants reported that the app failed to work at times. Technical problems did arise in the study, including operating system updates for both iOS and Android systems disabling the app for a short period of time. Parents who changed mobile phones during the study were required to contact the research team to obtain another code to access the app, which may have further reduced app usage. Furthermore, although push notifications were perceived to be relevant and timely, nearly 40% of participants were unsure how to retrieve push notifications once they had disappeared from the screen. This might explain the relatively low proportion (8%) of push notifications opened. Given that push notifications were the primary mechanism to drive parents to engage with the app content, this was very likely to have limited the dose of the intervention received and its subsequent impact on infant feeding behaviors and outcomes. This is further supported by app analytics data, which indicate that the use of the app did indeed decline over the duration of the study.

Understanding factors influencing engagement with mHealth programs and how these can be maximized over time is critical to program effectiveness. Our quantitative analysis of app usage [44] revealed higher engagement with the program among those recruited by their health practitioner, those who registered when their infant was younger, those who were first-time mothers, and those using both the app and website (via email links) compared with those using the app alone. This suggests that, to maximize engagement and potential impact, consideration should be given in the future to focus on recruiting first-time parents, with the help of health practitioners during the early postnatal or antenatal period. Future iterations of the program should include design features to improve access to push notifications and to maximize engagement over time using multiple methods (eg, email and push notifications) as well as the inclusion of more interactive features such as a forum and other tools to promote ongoing engagement. Technical issues also need to be addressed in a timely manner, including accommodating any operating system updates to ensure the program is functional at all times.

In addition to intervention dose, the lack of intervention effect may also be because of the sample size of this feasibility study, which was not powered to detect differences between groups but rather inform sample size calculations for a subsequent larger randomized controlled trial. The study was limited by the significant difference in a number of baseline characteristics between the study groups. Although baseline differences were controlled for in the statistical analysis, this reduces the power to detect differences in key outcomes between the groups.

The timing of the intervention may have contributed to the program’s limited impact on outcomes, particularly for breastfeeding. The average age of infants at the time of enrollment was 7 to 8 weeks, suggesting that the intervention missed the critical period for breastfeeding support, with national data indicating that 40% of mothers introduce formula by 1 month of age [14]. Research has also indicated that plans about whether a mother will breastfeed and for how long are made antenatally [45], and this was consistent with our qualitative findings [43] where mothers reported that plans for feeding their infant were made before enrolling in the program. This highlights the importance of commencing the program before birth and providing very early postnatal breastfeeding support to influence breastfeeding outcomes.

The GH program specifically targeted socioeconomically disadvantaged parents due to the socioeconomic disparities in obesity risk emerging in early infancy [17]. Our findings suggest that we had some limited success in reaching these parents, with just over half of mothers (51.6%) and nearly 70% of fathers not having a university-level education, with education commonly used as a proxy for socioeconomic position [46]. This is similar to the national average [47] but higher than that reported in other group-based obesity prevention trials in infancy. For example, the proportion of mothers without a university education was 46% in the InFANT trial [48] and 42% in the NOURISH trial [49]. However, a home-visiting trial in disadvantaged communities managed to recruit three-quarters of participants without a university education [50]. Surprisingly, we found no difference in the education levels of mothers when recruited by primary health care practitioners in disadvantaged communities and those recruited via social media [42]. It may be that primary health care practitioners were more selective in offering the program to less vulnerable parents. Our findings indicate the importance of catering for Android phone users if mHealth programs target socioeconomically disadvantaged parents, with higher Android phone usage among those without a university education compared with university-educated mothers in our sample. Given the greater need in socioeconomically disadvantaged parents, further research is required to ascertain how best to engage these parents in obesity prevention interventions and indeed whether mHealth programs provide a useful mode of delivery.

The use of mHealth for obesity prevention in early childhood is a rapidly growing field, with a number of trials underway [51-54]. However, we are unaware of any other studies reporting the outcomes of a mobile phone app targeting parents’ healthy infant feeding practices. A recent meta-analysis of 16 studies in developed countries has shown e-technologies (including SMS, Web, and interactive computer agent) to be effective in improving rates of breastfeeding initiation, duration, and exclusivity [55]. This review however did not include any studies utilizing a mobile phone app. Only one other published study [56] has reported the effectiveness of an mHealth program targeting infant feeding. That study [56], by Jiang et al in Shanghai, China, found that a weekly SMS from third trimester of pregnancy to 12 months postpartum resulted in a significantly higher rate of exclusive breastfeeding at 6 months and a significantly lower rate of the introduction of solid foods before 4 months. However, the intervention had no effect on other infant feeding practices, including taking a bottle to bed, drinking from a cup, or using food as a reward.

### Strengths and Limitations

The key strength of this study was that the intervention was informed by behavior change theory and extensive formative work and had high rates of retention. The use of a quasi-experimental study design was a limitation with a number of baseline differences between the intervention and the comparison groups; however, these differences were controlled for in the statistical analysis. There was also a likely selection bias in that the mothers who took part in both groups may have been more interested and motivated to achieve desirable infant feeding practices by virtue of their interest in this research despite having similar levels of education as national average [47]. This is supported by the high rates of exclusive breastfeeding at baseline (84% in BFF and 82% in GH compared with national average of 48% in infants less than 3 months of age [14]) and low proportion introducing solids early (13% at 4 months in both groups compared with national average of 35% [14]). This may have limited the ability to detect intervention effect. A randomized recruitment strategy with a more representative sample of mothers might offer more potential to show improvements in infant feeding practices. Measurement of infant feeding behaviors based on self-report in both groups was no doubt subject to social desirability bias. Parental report of weight and length may be subject to transcription errors and would be more accurately collected by investigators.

### Conclusions

Our study design was feasible in that there was an excellent retention rate over time for participants who completed the enrollment survey, and the results provide a useful estimate on which to base a sample size calculation for a larger study. An mHealth intervention using a smartphone app to promote healthy infant feeding behaviors is a feasible and acceptable mode for delivering obesity prevention intervention to parents but further work is required to sustained engagement and use over time. The limited impact of the program on key measureable infant feeding outcomes may reflect that some participants received a low intervention dose because of unforeseen technical problems, the timing of the program, participant selection bias, and/or limitations in the study design. It is recommended that mHealth programs targeting infant feeding commence antenatally and future iterations of the program have contingencies in place to address technical issues in a timely manner and design features to maximize engagement over time. Future research using larger randomized controlled trial designs are required to determine the effectiveness of mHealth programs for obesity prevention in infancy.

## Appendix

Multimedia Appendix 1 Mixed effects polynomial regression model for BMI z-score, weight, and length trajectories.

## Abbreviations

AOR: adjusted odds ratio
BFF: Baby’s First Food
BMI: body mass index
GH: Growing healthy
SMS: short message service
