# Supplementary material for: Key Lessons and Impact of the Growing Healthy mHealth Program on Milk Feeding, Timing of Introduction of Solids, and Infant Growth: Quasi-Experimental Study
Source: JMIR Mhealth Uhealth. 2018 Apr 19;6(4):e78. doi: 10.2196/mhealth.9040 (PMC5934537; doi:10.2196/mhealth.9040)
Supplement: Multimedia Appendix 1 [file mhealth_v6i4e78_app1.pdf]

Supplementary table 1. Mixed effects polynomial regression model for BMIz, weight and length trajectories

	BMIz			Weight			Length		
	β	Std. Err.	P-value	β	Std. Err.	P-value	β	Std. Err.	P-value
Age	2.10	0.08	0.00	0.98	0.02	0.00	3.91	3.77	0.00
Age^2	-0.78	0.03	0.00	-0.04	0.00	0.00	-0.19	-0.20	0.00
Age^3	0.09	0.00	0.00	-	-	-	-	-	-
GH	-0.08	0.08	0.31	-0.04	0.05	0.43	-0.14	-0.58	0.53
Gender	0.06	0.08	0.48	0.24	0.04	0.00	1.41	0.98	0.00
Whether first born	-0.11	0.08	0.20	-0.03	0.05	0.55	0.12	-0.34	0.61
Dummy use	0.12	0.08	0.14	-0.07	0.05	0.11	-0.57	-1.01	0.01
Maternal smoking status	-0.08	0.19	0.67	-0.31	0.10	0.00	-1.52	-2.54	0.00
Maternal country of birth	0.04	0.13	0.73	0.05	0.07	0.50	0.02	-0.68	0.96
Maternal education	-0.06	0.06	0.29	-0.06	0.03	0.05	-0.25	-0.56	0.12
Paternal education	0.05	0.06	0.41	0.01	0.03	0.69	-0.04	-0.36	0.83
Maternal employment status	-0.01	0.12	0.94	-0.03	0.07	0.66	-0.40	-1.05	0.22
Paternal employment status	0.01	0.23	0.98	-0.14	0.13	0.26	-0.31	-1.52	0.62
Household income	0.05	0.04	0.25	0.03	0.02	0.19	0.09	-0.13	0.43
Maternal prepregnancy BMI	0.01	0.01	0.12	0.01	0.00	0.03	0.04	0.00	0.06
Maternal age	-0.01	0.01	0.58	0.00	0.01	0.38	-0.02	-0.07	0.58
Intercept	-0.03	0.48	0.95	3.30	0.27	0.00	49.12	46.54	0.00
Random-effects Parameters									
Variance(Age)	0.45	0.05		0.26	0.02		0.76	0.11	
Variance(Age^2)	0.05	0.01		0.02	0.00		0.08	0.02	
Variance(intercept)	0.64	0.06		0.36	0.02		2.06	0.13	
Covariance (Age, Age^2)	-0.96	0.01		-0.92	0.01		-0.84	0.05	
Covariance (Age, intercept)	-0.21	0.12		0.09	0.10		-0.33	0.11	
Covariance (Age^2, intercept)	0.11	0.14		-0.05	0.11		0.21	0.14	
Variance(Residual)	0.90	0.03		0.34	0.01		1.95	0.07	
Log likelihood	-2360.33			-1339.17			-3777.31		
